# Overview of lunar dust toxicity risk

**DOI:** 10.1038/s41526-022-00244-1

**Published:** 2022-12-02

**Authors:** Michael Pohlen, Danielle Carroll, G. Kim Prisk, Aenor J. Sawyer

**Affiliations:** 1 Stanford University, Stanford, USA; 2 University of California, San Diego, USA; 3 University of California San Francisco, San Francisco, USA; 4 Translational Research Institute for Space Health (TRISH), Houston, USA; 5 Eastern Virginia Medical School, Norfolk, USA; 6 UC Space Health, University of California, San Francisco, USA

**Keywords:** Risk factors, Respiratory signs and symptoms, Acute inflammation, Iron, Corneal diseases

## Abstract

Lunar dust (LD), the component of lunar regolith with particle sizes less than 20 μm, covers the surface of the Moon. Due to its fineness, jagged edges, and electrostatic charge, LD adheres to and coats almost any surface it contacts. As a result, LD poses known risks to the proper functioning of electronic and mechanical equipment on the lunar surface. However, its mechanical irritancy and chemical reactivity may also pose serious health risks to humans by a number of mechanisms. While Apollo astronauts reported mild short-lived respiratory symptoms, the spectrum of health effects associated with high-dose acute exposure or chronic low-dose exposure are not yet well-understood. This paper explores known and potential human risks of exposure to LD which are thought to be important in planning upcoming lunar missions and planetary surface work.

## Introduction to lunar dust

Since the late 1950s, marked by the deployment of the first Soviet Luna probes and the subsequent Apollo missions, humans have actively studied the lunar regolith comprised of loose deposits of rock covering the surface of the Moon. For over four billion years, this layer of rock and soil composed mostly of oxygen, silicon, and iron has formed from meteorite impacts fracturing the lunar bedrock. No constant weathering processes analogous to those caused by wind, rain, ice, or biological activity alter the lunar regolith, though solar wind and cosmic rays, in addition to the mechanical weathering of impact events, do alter the geology and chemistry of the lunar surface over time and slowly reduce average particle size^1^. Lunar regolith ranges in size from large boulders to ultrafine dust, with most particles measuring less than 1 cm in size. Lunar soil refers to grains less than 1 cm in size, while the finest regolith components, those particles with a diameter under 20 μm, are referred to as lunar dust (LD)^2^. The respirable fraction of lunar dust, like other particulate matter, is generally divided into ultrafine <0.1 μm, fine 0.1–2.5 μm, and coarse 2.5–10 μm fractions. This dust comprises approximately 20% by weight of the lunar surface soil samples returned to Earth by Apollo missions^3^. The shape and surface of these LD particles vary significantly. Apart from small, glassy agglutinates formed from the melting of lunar soil particles by the heat of meteoroid impacts, most larger particles of LD have sharp, jagged edges, having been fractured but not melted as a result of repeated impacts^4^. These particle surfaces can remain irregular for extended periods of time due to the lack of consistent weathering. Their large surface area and exposure to galactic and solar radiation likely increase the surface reactivity of the particles by creating large numbers of dangling chemical bonds and unsatisfied electron valences^5^. In addition, iron in the Fe^0^ valence state (often referred to as nanophase iron) present in the particles may contribute to the formation of reactive oxygen species when in contact with human tissue, though the likelihood and severity of this possibility are unknown^5–8^. While this dust poses known risks to the proper electrical and mechanical functioning of spacecraft and equipment sent to the lunar surface, questions remain regarding the possible harms to human health via both the mechanical and chemical irritant properties of LD^9^. Given the known deleterious effects of exposure to terrestrial particulate matter as well as some mineral dusts such as quartz, concern exists for the potential of acute and/or chronic multiorgan toxicities^10–12^. The following sections discuss these potential risks using references collected from the PubMed and Scopus databases using the key words “lunar dust,” “lunar dust toxicity,” and “silicosis,” as well as references previously known to the authors.

## Topic review

### Historical exposures

Human exposure to LD began in 1969 with the extravehicular activities (EVA) performed by the crew of Apollo 11 and continued through five additional Apollo missions over the subsequent three years. Over a dozen EVAs were performed, and a large quantity of lunar regolith was returned to Earth for study, both by these Apollo missions and by Luna probes. Following each EVA, a substantial amount of LD was introduced into the lunar module through adherence to the cloth outer layer of the spacesuits, aided by the static charge. Numerous astronauts reported ocular and respiratory symptoms after these exposure events despite attempts to remove dust using brushes and vacuum systems inside the cabin in later Apollo missions^13^. Specifically, they reported erythematous, watery eyes with decreased vision as well as respiratory symptoms such as throat irritation and cough^14^. One flight surgeon who came in contact with LD after capsule return reported what may have been an allergic type IV hypersensitivity reaction that worsened with each successive exposure to the LD^14^. In all cases, these symptoms were transient and improved within 24 h of onset. Exposures were never associated with symptoms significant enough to cause mission failure, though anecdotes provided by astronauts suggest that performance was impacted. Unfortunately, concentrations of LD present in the spacecraft after EVA were never measured. No comprehensive assessment of LD toxicity was performed in the decades following the Apollo missions, though follow-up pulmonary function testing after the Apollo missions demonstrated no long-term adverse effects to the respiratory system in these astronauts, and engineering controls have minimized exposure to humans that have handled material from the lunar surface^15,16^.

### LADTAG & permissible exposure limits

**Fig. 1:**
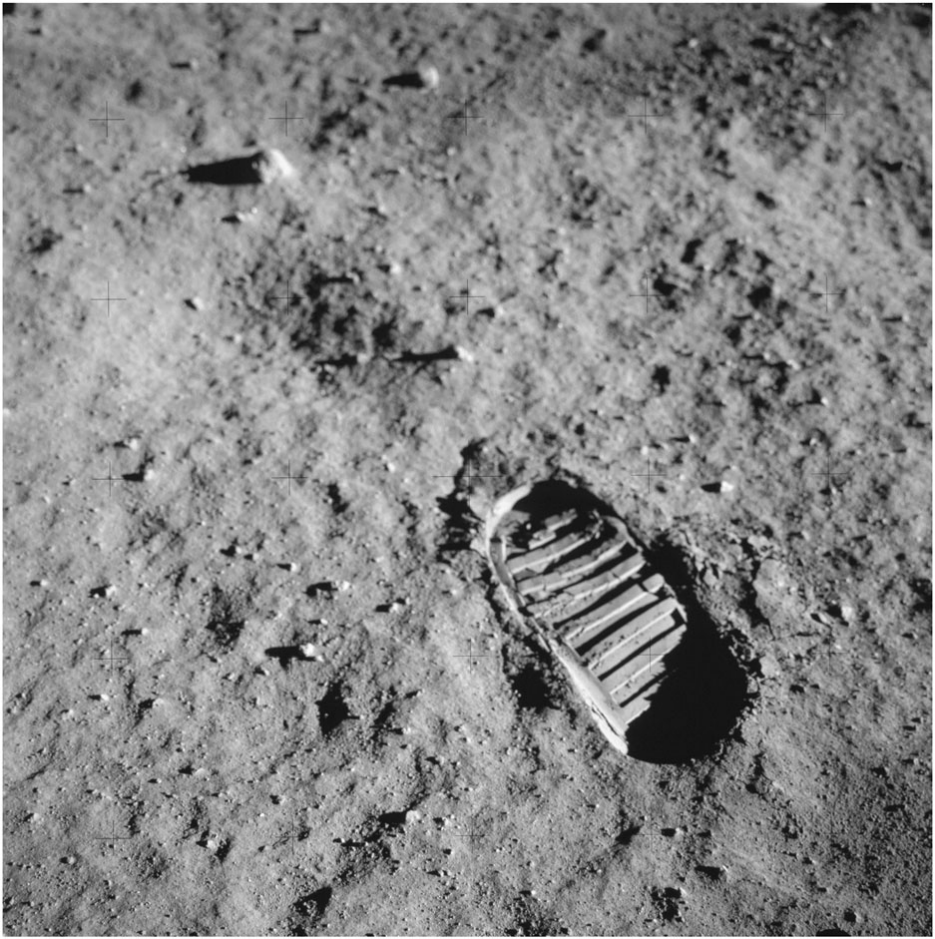
Footprint in lunar regolith. The footstep of Buzz Aldrin compacting fine grains of lunar soil during an Apollo 11 EVA^81^. *Copyright © NASA Public Domain*.

**Fig. 2:**
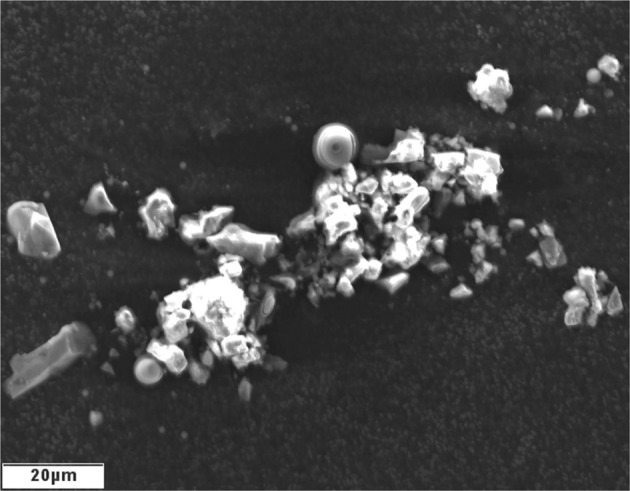
Lunar dust grains. Scanning electron microscopy images of native lunar dust grains collecting during an Apollo 17 EVA^82^. *Copyright © NASA Public Domain*.

**Fig. 3:**
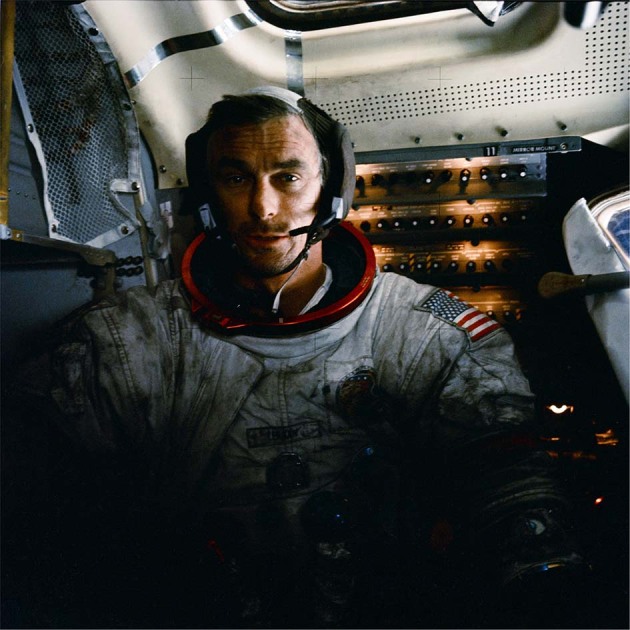
Lunar dust adherent to spacesuit. Gene Cernan, Apollo 17 commander, is seen with his spacesuit covered in lunar dust following an EVA. Lunar dust can also be seen adhering to the interior of the lunar module^83^. *Copyright © NASA Public Domain*.

As evidence of the dangers posed by terrestrial mineral dusts and particulate matter grew in the 1970s through 1990s, attention returned to the possible adverse health effects of LD exposure^17,18^. In 2004, U.S. President George W. Bush announced a planned manned return to the lunar surface by 2020, prompting NASA to urgently examine the need to develop permissible exposure limits (PELs) for LD on future long-duration missions. NASA formed the Lunar Airborne Dust Toxicity Advisory Group (LADTAG) to accomplish this goal, tasking it with funding and conducting a series of investigations into unresolved questions regarding the noxious potential of LD. Following completion of these studies, LADTAG released its final report in 2014 and recommended a preliminary LD PEL of 0.3 mg/m^3^ for a 6-month lunar mission with eight hours of LD exposure for five days per week^19^. This number was based on experimental evidence examining both inhalational and ocular toxicity using pulverized lunar regolith and is toward the lower end of the range of PEL estimates (0.2 mg/m^3^ to 0.7 mg/m^3^) derived from multiple studies with varying methodologies. This value of 0.3 mg/m^3^ is similar to that developed for crystalline quartz, with a PEL of and 0.1 mg/m^3^, versus a lower toxicity material, titanium dioxide, with a PEL of 5 mg/m^3^. NASA has accepted and adopted this preliminary PEL as a design standard for upcoming lunar missions and is exploring ways to best monitor cabin air quality to ensure that LD concentrations within the spacecraft do not exceed these exposure limits. Of note, while the preliminary PEL is based on exposure occurring five days per week, current spacecraft will likely result in exposure every day given there is no mechanism for LD avoidance. The following sections describe the pathophysiology of LD toxicity, research projects supported by LADTAG, and the studies performed since the release of the report (Figs. 1–3).

### Inhalational toxicity

The toxic effects of inhaled particulate matter, and the hypothesized toxic effects of LD, are determined by several key characteristics of the particles themselves, including form, surface reactivity, biopersistence, particle size, solubility, and composition, among others, with size most influential in determining the location of particle deposition in the respiratory tract^20^. Toxic mechanisms of action influenced by these characteristics are complex for a variety of reasons, including the ability of a particle to act in multiple steps of a pathogenic process, remain intact within the body for an extended period of time, and be modified by biochemical processes in vivo^5^. The general inflammatory pathway by which particulate matter is thought to induce adverse effects is most applicable when inhaled, but also is believed to apply via a similar mechanism when skin or soft tissue absorbs or is penetrated by particles. In the lung, particulate matter enters the air spaces and is first coated by endogenous molecules, most notably proteins; it is thought to react with antioxidants present in the surfactant and fluids lining the alveolar surface^5,21^. The particles are then cleared via either the mucociliary escalator in the conducting zone or via phagocytosis by macrophages within the alveoli. These mechanisms have very different time scales with mucociliary clearance occurring in hours to days, while clearance by alveolar macrophages takes months to years^22,23^. In the latter pathway, the extended presence of the particle, even after macrophage phagocytosis with subsequent apoptosis or lymph node migration, can prompt the release of oxidants, cytokines, and growth factors and sets off an inflammatory cascade with the recruitment of additional immune cells (to include neutrophils and new alveolar macrophages). The uncleared particles can be absorbed repeatedly by successive generations of macrophages, leading to chronic inflammation^24^. The particles may also directly damage the cells lining the alveoli, allowing nanoscopic particles to enter the bloodstream and, eventually, reach other target tissues. In addition, the interaction of cellular reactive oxygen species with free radicals is thought to constitute one of the most concerning mechanisms of direct toxicity^25^. Any chronic inflammation and oxidative stress may contribute to the development of a variety of pathologic pulmonary conditions, including cancer and COPD, if permitted to continue for a sufficient length of time.

LD is hypothesized to possess deleterious effects similar to these other types of particulate matter given similarities in composition and size, though the aforementioned shape, surface reactivity, and biopersistence of LD are incompletely described^5^. In addition, the potential adverse effects caused by possible olfactory, pulmonary, and gastrointestinal intake and subsequent vascular and lymphatic transport of these nanoparticles to other target organs such as the central nervous system and heart are numerous and not fully understood^26–28^. However, this presumed pathophysiological pathway of LD toxicity is similar to and partially derived from the effects of other specific types of inhaled particulate matter, such as particulate dust that contains crystalline silica, a widely-studied compound responsible for the development of silicosis. Silicosis is an occupational lung disease that results when respirable dusts containing certain types of silicate material, such as crystalline quartz, lodge in the alveoli of lungs, thereby setting off an inflammatory cascade that ultimately leads to fibrosis^29^. The aforementioned cascade is mediated by macrophage phagocytosis and lysosomal damage, a process that may parallel what occurs with respirated LD. Silicosis progresses even after exposure to silica ceases, and no curative therapies currently exist. Given the dissimilarities in chemical composition and questionably reactivity, the extent to which silicosis may serve as a model for the toxic effects of LD is unclear, though LD exposure is thought to be less hazardous than crystalline quartz at similar concentrations based on the studies performed by LADTAG.

Given the aforementioned concerns regarding several mechanisms by which particulate matter, and by extension LD, may cause adverse effects, the main thrust of the research efforts supported by LADTAG was aimed at characterizing the inhalational toxicity of LD. Unlike many prior studies, these investigations utilized small quantities of authentic LD collected by Apollo 14 rather than a simulant dust. However, sufficient quantities of native LD were not available to facilitate subsequent inhalational trials, so the properties (size, composition, etc.) of native, unground dust were compared to ball-milled and jet-milled LD to determine if either milling process could convert larger lunar regolith particles to respirable-sized LD grains similar to that generated on the lunar surface^30^. Jet-milled dust, formed by collisions between particles of lunar soil rather than between lunar soil and another object, was determined to be most similar with respect to its process of formation and was therefore used in future trials where native unmilled dust was unavailable. James et al. then compared molecular and cellular markers of toxicity after exposure of rats to known concentrations of ball-milled LD, jet-milled LD, native unground LD, TiO_2_, and quartz via intratracheal instillation, a surrogate for inhalation^15^. This was done in order to comparatively model benchmark doses of LD using the known PELs, safe exposure estimates (SEE), and relative toxicities of TiO_2_ and quartz, thereby determining an estimated safe exposure estimate in mg/m^3^ for LD that can be used to determine a PEL. The recommended safe exposure estimates for episodic exposure to LD on 180-day missions ranged from 0.3 to 0.5 mg/m^3^ depending on preparation of LD. The authors also found that the three preparations of LD were toxicologically similar and that the milling of the LD did not increase reactivity. Given the lack of increased reactivity with milling intended to simulate the lunar environment, the reactivity of LD on the lunar surface remains unclear.

A second LADTAG study, conducted by Lam et al., estimated PELs via a different approach, examining the cellular and histopathologic changes observed in rats after one month of episodic inhalation of LD at varying concentrations^31^. This was conducted in order to determine a no observed adverse effect level (NOAEL) to be used as the toxicological point of departure. The investigators found that at an inhaled concentration of 6.8 mg/m^3^ no adverse effects were observed. A slight increase in neutrophils was noted at this concentration but determined to be an adaptive response rather than an adverse effect. The next concentration tested, 20.8 mg/m^3^, was associated with only mild adverse effects, including neutrophilic inflammation, lymphoid hyperplasia, and granulomas, without evidence of fibrosis at the 3-month endpoint. Extrapolating these results from a rat model to human and from a 1-month exposure to a 6-month mission produced an estimated SEE of 0.3 mg/m^3^. A third study, conducted by Scully et al., utilized the same data of rat inhalational exposures but applied an alternative method to establish a SEE^32^. They created a range of points of departure based on benchmark dose modeling by determining estimated dose-response curves for the experimental endpoints that appeared to be sensitive to dose. The estimated benchmark dose and lower 95% confidence level of the benchmark dose resulted in SEEs of 0.7 mg/m^3^ and 0.2 mg/m^3^, providing reliable upper and lower bounds to a range of possible SEEs. Because the SEEs determined by the above three methods, two of which were entirely independent, were relatively similar, LADTAG voiced confidence in the validity of their datasets and elected to conservatively set the recommended preliminary PEL at 0.3 mg/m^3^.

### Potential pulmonary toxicities in need of further investigation

While the LADTAG report provides a PEL for pulmonary and ocular toxicity under a certain set of mission circumstances, numerous open questions remain. While the surface reactivity of LD in situ has long been difficult to assess after sample return to Earth, evidence indicates that simulating the activation of LD by radiation and meteorite bombardment by milling LD does not increase the ability to generate reactive oxygen species^19^. In addition, it is theorized that any increased reactivity resulting from activation on the lunar surface is rapidly neutralized by the moist, oxygenated environment of the spacecraft. However, studies by Wallace have suggested that the surface chemical reactivity to ground lunar material may persist for several hours following exposure to air. Whether these chemical measurements translate to corresponding increases in toxicity, and whether ground lunar material is representative of actual LD on the lunar surface, is at present unknown^6^. While this no doubt requires further study, LADTAG determined that any increased surface reactivity is likely less important than direct toxicity with respect to adverse human health effects^33,34^. In addition, questions have been raised regarding the ability to properly model the effects of microgravity or reduced gravity on the deposition of fine LD within the lung. However, investigations conducted in parabolic flight using aerosolized polystyrene latex particles measuring 0.5 to 1.0 μm have suggested that while reduced gravity reduces the total quantity of dust deposited in the lung, the site at which the remaining deposition occurs is more distal^35^. This raises the possibility of greatly increased residence times for the dust if it deposits beyond the reach of the mucociliary clearance system.

While most of the aforementioned studies have examined the effects of long-term exposure to what may represent low concentrations of LD, few have examined shorter exposures to higher concentrations. The known acute exposures of Apollo astronauts to LD did not produce identifiable long-term sequelae, though the exposures were brief and occurred intermittently over a period of only several days, with additional uncertainty as to dose due to absence of cabin concentration measurements. Current data demonstrate no long-lasting respiratory symptoms in response to single high-dose exposures to terrestrial volcanic ash, though asthma exacerbations and episodes of bronchitis are often triggered^9,17^. Much less is understood about the acute complications of repeated high-dose exposures to LD-like material. Though it is expected that inhaled LD will trigger a cytokine-mediated inflammatory cascade, a process also observed in acute volcanic dust exposure, whether the effects will include chronic fibrotic changes is unknown^36^. Repeated high-dose exposures could potentially produce more acute lung disease via irritant and/or allergic pathways, requiring in-mission management to avoid significant disability or death. Though its chemical composition varies from LD, there are reported cases of acute respiratory distress syndrome (ARDS) with inhaled cement dust in otherwise healthy individuals, an outcome that in a space environment would likely prove fatal^37^. While unlikely to occur as a result of regolith exposure, this would certainly be a catastrophic outcome in a profoundly resource-limited environment such as the lunar surface. Furthermore, while less common than its chronic form, acute silicosis may produce symptoms within weeks to months after sufficiently large exposures and lead to life-threatening complications within a year^38,39^. Severe isolated allergic responses may be also possible^31^. In total, the acute effects of numerous high-dose LD exposures over a period of weeks to months are undescribed and will require close future study.

One large additional concern pertains to the applicability of the LADTAG for lunar missions to regions of the lunar surface with geology or conditions dissimilar to those at the Apollo 14 landing site where LD samples were gathered. The soil samples tested thus far include a mixture of mare and highland soils near the surface of the layer of regolith. However, future lunar missions may enter polar regions where portions of the surface of craters are never exposed to sunlight. In addition, future missions may mine lunar regolith or bedrock for water ice or minerals. It is unknown if LD mined from areas beneath the near surface or in permanently shadowed regions poses an increased risk to human health, though geographic variation in the toxicity of terrestrial dusts has been observed^40^. While much focus has been given to LD, such studies must also be repeated for potential exposures to particulate matter from the surface of other exploration targets, including asteroids or Mars, as their chemistry and geology vary significantly from that of the moon.

### Ocular toxicity

Given astronaut reports of eye irritation following exposure to large quantities of LD, NASA sponsored in vitro and in vivo ocular testing with authentic LD to determine toxicity^41^. In vitro assay testing suggested LD possesses minimal chemical irritancy and reactivity. Follow-up in vivo Draize testing in a rabbit model with native LD confirmed this finding and suggested only minimal associated mechanical irritancy. Slight conjunctival erythema and edema were observed with large doses of LD, specifically 70 mg of lunar dust, but these effects disappeared within 24 h of cessation of exposure, and no corneal abrasions were noted. As a result of this study, LADTAG labeled the dust as merely a nuisance and recommended against special protective precautions unless the astronaut independently finds the dust to be irritating^41^. Though unlikely, should ocular LD exposure be sufficient to cause eye injury such as a corneal abrasion, fluorescein strips would be useful in the diagnosis of any corneal injury. While ocular irritation may be transient in studied scenarios, repeated donning and doffing of spacesuits covered in LD might make this exposure a frequent occurrence, and attention will be required to ensure that the effects of this hazard do not compound over time.

### Dermal effects

Lunar dusk is known to be highly abrasive and so clothing contaminated with LD has the potential for skin abrasion, especially in the confines of a rather rigid spacesuit. Skin abrasion irritates the dermal/water-vapor barrier (dermis), leading to dermatitis and/or sensitization of the skin. A transdermal-impedance technique has been employed to measure the abrasive effect of simulant LD on pig skin, a high-fidelity model for human skin^42^. The skin was abraded with the simulant JSC-1A to test the methodology, and the effect was measured using transdermal impedance. This provided a measurement of damage to the dry, outermost layer of the skin, the stratum corneum, which is important for the barrier function of the skin. The preliminary results of these studies showed that JSC-1A had abrasive properties akin to commercial sandpaper^42^. Further investigations such as skin toxicology studies, including chemical irritancy evaluation and sensitization tests with native lunar dust, were not performed.

### Immunologic considerations

A discussion of the effects of LD on the human body would be incomplete without addressing possible immune system dysregulation in the altered-gravity environment. Some recent studies suggest the global dysregulation of adaptive immunity and heightening of certain aspects of innate immunity, generating an immunologic landscape that differs from that seen on Earth^43–45^. Others have suggested minimal if any broad impairment, including the NASA twins study^46^. With this in mind, understanding and predicting immune system effects of chronic LD exposure pose a particular challenge. In vivo studies performed on mice have demonstrated persistence of a proinflammatory state beyond that seen in Earth-based controls; increased cytokine production over a longer period of time appears to be responsible for the impairment of normal induction of antigen-specific immune tolerance^43^.

Furthermore, reactivation of latent viruses, including herpesvirus, with associated viral shedding has been observed in astronauts during and after both shuttle and ISS missions. While postflight viral shedding detected in urine and saliva samples is typically subclinical in this patient population, in certain cases, live viruses identified on tissue culture have been associated with dermatologic conditions such as atopic dermatitis and other skin lesions, both inflight and following return to Earth^47^. The potential for acceleration or exacerbation of these conditions by chronic LD exposure will need to be defined in order to better understand potential immunologic implications of long-duration lunar missions; in the near term, however, screening for infectious diseases such as herpes and tuberculosis pre-flight may be implemented or expanded. Furthermore, it is unclear how presumed changes in lymphatic flow might contribute to some of the microgravity-associated physiologic changes that have been observed; further study in this area is also necessary.

### Possible other end organ toxicities & future areas of investigation

The effects of LD on numerous other physiologic systems have yet to be characterized. Chief among these unexplored effects of LD exposure is possible cardiovascular (CV) toxicity. An increasing number of studies suggest that particulate matter exposure increases CV morbidity and mortality, but uncertainty remains as to the exact mechanism(s) of disease and which components of particulate matter most contribute to these effects^48^. It is unknown if LD particles may contribute to a similar increase in negative CV outcomes, and studies have been proposed to measure biomarkers associated with cardiovascular disease after intratracheal instillation of LD in a rat model^19^. Deleterious effects on cardiac or vascular health may be compounded by known physiologic changes in spaceflight as a result of microgravity, including cardiac deconditioning, dramatic fluid shifts, and an increase in the frequency of transient arrhythmias^49^.

Though no gastrointestinal toxicity has yet been demonstrated, the size, nature, and composition of the dust granules raise concern for potential long-term sequelae associated with chronic exposure. Research conducted over the course of the last decade has established an understanding of the permeability of oropharyngeal, gastric, and gut mucosa to nanoparticulate matter^50^; while this may sometimes prove advantageous in the controlled, terrestrial environment by prompting new therapeutic approaches to target a variety of pathologic gastrointestinal conditions, it may pose challenges in the remote, expeditionary lunar setting. Oral health effects of chronic LD ingestion have not yet been explored, such as any possible contribution to the development of gingivitis or the ability to erode tooth enamel due to its abrasive nature. As invasive dental care measures are unlikely to be available on early missions, any effects may pose significant threats to overall crew health and, consequently, effective mission completion. Terrestrially, ingestion of nanoparticles and subsequent deposition of chemical components into the cellular cytoplasm has already been tied to the development of conditions such as Crohn’s disease and colon cancer^51^, though the precise mechanisms in play remain unclear. In addition, the presence of iron in the makeup of these nanoparticles raises the concern for iron toxicity with prolonged exposure to high volumes of LD.

As the microbiome in its entirety remains not fully understood, further concerns have been raised regarding potential unwanted alterations to the bacterial populations colonizing the gut; recent studies suggest that the high availability of iron in the gastrointestinal tract may enhance expression of virulent factors in pathogenic bacteria^52^. In particular, proliferation and activity of the bacterium *Escherichia coli* is facilitated by the presence of iron in the colonic lumen, suggesting enhanced pathogenicity in the setting of unregulated iron availability. The iron in lunar regolith is in an electrochemically reduced state, as compared to the oxidized state of iron in most chemical compounds on Earth; it is unclear how this may further affect iron absorption and metabolism. A generalized inflammatory state within the gut may predispose crewmembers to malabsorption not only of iron and other nutrients, but also of water, yielding a mixed gastroenteritis-type dehydration effect. As chronic inflammation is known to cause progressive tissue fibrosis, long-term, unregulated exposure to LD could reasonably lead to permanent tissue changes affecting the absorptive capabilities of the gut lumen.

In addition to these multisystem toxicities, there is concern for the potentiating effects of exposure on other spaceflight-associated hazards and for possible end organ toxicities not yet identified. For example, LD may compound the harmful consequences of increased radiation exposure and microgravity, or these alterations in the spaceflight environment may exacerbate the negative effects of LD exposure. Among less explored potential toxicities, concern exists for neurological consequences of exposure as a result of multiple studies examining the direct cytotoxic effects of simulant LD, though no comprehensive studies have yet confirmed such findings with native LD^33,53^. In addition, evidence exists for particulate matter-associated mild cognitive impairment in the elderly, though the exposure time and composition of the particulate matter in this dataset differs from that of expected astronaut LD exposures^54^. How any such toxicity would interact with the damage to CNS structures and peripheral nerves as a result of fluid shifting and increased radiation exposure is also unclear. Much like any potential nephrotoxicity, LD-induced chronic kidney disease may be compounded by renal pathologies known to occur with high frequency during spaceflight, such as nephrolithiasis^55^. A much longer-term difficulty for a self-sustaining lunar outpost may be the impact of LD on reproductive and developmental health via decreased fertility and a higher incidence of birth defects, though the impact of radiation is likely to have a more directly deleterious effect^56,57^. The various hypothesized toxicities are potential peripheral effects of LD inhalation and its translocation from the lungs^27^. Further studies are required to substantiate these risks.

### Countermeasures: exposure prevention & reduction

Numerous countermeasures have been proposed to combat the threat of LD toxicity, including efforts to passively and actively prevent entry and remove dust from habitable spaces. On Apollo missions, nylon bristle brushes were utilized to remove larger dust particles from suit and visor exteriors before reentry into the lunar module^9^; wet wipes were used for surface cleaning inside module. In later missions, vacuum cleaners and cabin air filters scrubbed LD from the air inside the lunar module and were met with mixed success, per astronaut reports. Though these efforts to reduce dust were noted to be moderately effective, longer-duration lunar missions will require more robust deterrents to maintain proper equipment functioning and avoid adverse crew health effects.

Numerous additional methods have also been proposed. First, dust may be prevented from entering equipment and adhering to spacesuits by using electrostatic or electrodynamic shielding to repel dust away from surfaces^58^. The dust component of lunar regolith, as a result of the constituent nanophase iron, is sufficiently magnetic to be lifted off or repelled from a surface with a simple handheld magnet^59^. To further reduce adhesion, objects exposed to LD should ideally possess non-pliable, non-polar surfaces to reduce the ability of LD to adhere and embed, with attention paid to designing specific surface topographies to assist in this effort^60^. Gels, liquids, or pressurized gases could also be used to mechanically remove LD from surfaces^61^. Consideration has been given to a suitlock or suitport to be installed to enter and exit spacesuits directly from the cabin and/or rover, thereby preventing the dust-laden exterior of the spacesuit from entering the spacecraft^62^, although this raises issues with suit maintenance. Multi-stage HEPA air filters can be used to clear cabins of suspended LD, with magnetic filters added to remove dust by attracting the constituent nanophase metallic iron present in particles^63^. Lastly, despite these prevention methods, should large amounts of LD be introduced into the habitat, personal protective equipment could be worn until LD concentrations can be reduced to acceptable levels. However, as the Apollo experience showed, some degree of crew exposure to LD is an almost inevitable consequence of Lunar surface activities.

### Countermeasures: exposure monitoring and diagnosis of toxicity

While efforts to prevent dust exposure may be effective in significantly reducing LD concentrations in habitable areas, it seems likely that exposures will still occur. This will require methods of monitoring LD concentrations as well as monitoring astronaut health regularly, diagnosing any pulmonary or other end organ toxicity early in order to more aggressively limit further exposure. An important first step will be to establish low-cost, low-weight, and high-accuracy methods of determining LD concentrations within the spacecraft or habitat at regular intervals and comparing them against the established PEL. This may be achieved using photometric or, less likely, gravimetric measurements, with instruments as small as handheld units in the case of the former, such as those utilized previously in LADTAG experiments^31^. Automated dust monitoring units, such as those used near construction sites, would likely save time and, once properly calibrated, provide critical data real-time data regarding exposure levels. A key challenge will be adapting terrestrial dust and particulate matter monitoring units to the size and morphology characteristics of LD. In addition, active monitoring of the reactivity of LD may prove critical, and designs for small fluorescence-based sensors have been developed to serve this purpose^64^.

While the task of monitoring LD concentrations may prove straightforward, monitoring the physiologic response to LD exposure may prove more challenging, especially in the resource-limited setting of long-duration spaceflight. Ideally, pre- and post-flight clinical, laboratory, and possibly imaging assessments of lung, cardiac, and other end organ structure and function would be used to monitor for onset of significant LD toxicity. For pulmonary toxicity, the area of greatest concern, clinical symptoms such as cough, shortness of breath, and fatigue may occur within months to years for acute toxicity but, if similar to silicosis, begin potentially decades after exposure in the case of chronic disease^65,66^. Exam findings such as crackles are unlikely to arise before the onset of symptoms, and imaging findings may also lag exposure by several years (though often arise before symptoms), such that pathologic diagnosis via bronchoalveolar lavage or lung biopsy may be the earliest indicator^67^. These procedures are essentially incompatible with performance during a lunar mission.

Inasmuch as the onset of symptoms in chronic toxicity would seem likely to occur well after the completion of a 6-month lunar mission, monitoring for adverse effects of LD exposure during the mission will be difficult. No serum markers have been identified that are sensitive and specific for the diagnosis of most occupational lung diseases, silicosis included, though a complete blood count and infectious and immunologic workup would likely assist in excluding other diagnoses. Nonspecific serum markers of inflammation and tumor biomarkers might have utility in screening and/or trending levels of disease severity, though they would help little in ascertaining the diagnosis or determining the level of functional impairment^68,69^. Serial pulmonary function tests (PFTs), including resting oxygen saturation, spirometry pre- and post-bronchodilator, and perhaps diffusing capacity of the lungs for carbon monoxide, may be more useful in measuring acute responses to LD exposure. Worsening PFTs in silicosis correlate with deterioration in imaging findings, with PFTs generally showing mixed obstructive and restrictive respiratory impairment in chronic disease^66,70^. Point-of-care PFTs may have a role in future lunar missions, as frequent measurements of pulmonary function would both help monitor for the onset of any acute LD toxicity and assist in the identification of any hypersensitivity reaction to LD. Small handheld spirometry units that interface with computers and mobile devices could be utilized at designated intervals to screen for changes in pulmonary function^71^. A potentially useful supplemental approach to PFTs is to actively monitor airway inflammation via NO concentrations in exhalate, as NO increases with worsening airway inflammation^5^. Such monitoring has been used in studies on the ISS, so the technical capability exists, although the extent to which LD exposure increases detectable airway inflammation is unclear^72^.

An even higher degree of uncertainty exists for other breath tests recently developed or under development, including exhaled breath condensate pH and exhaled breath temperature. In addition to measuring proxies for inflammation in exhalate, sputum analysis may also prove fruitful. Limited evidence suggests that sputum IL-1beta as well as inflammatory cell counts, including neutrophils and eosinophils, were correlated with rate of decline in pulmonary function in asbestosis and silicosis^73^. Should the inflammatory pathway of any LD toxicity mimic either pathology, in-mission laboratory examination of sputum markers, cell counts, and even transcriptome may detect early signs of functional impairment if analysis equipment could be made available.

Lung imaging in spaceflight is likely to prove more difficult than lung function evaluation and laboratory testing. Currently, the only imaging modality tested and used extensively in spaceflight is portable ultrasound^74^. While pulmonary ultrasound has proven effective in the emergency setting for identification of pneumothorax, pulmonary edema, consolidations, and diseases of the thoracic wall, the pneumoconioses, silicosis included, are not associated with any characteristic ultrasound pattern, and pathologic changes are difficult to detect until late- stage disease when pleural involvement is significant^75^. Should pulmonary LD toxicity present similarly, ultrasound is unlikely to play a significant role in early diagnosis, as distinctive pathology such as subpleural nodules are likely to form only after significant exposure.

### Countermeasures: treatment of toxicity

Although methods of primary and secondary prevention may be able to limit the development of LD toxicity, established disease, if similar to known pneumoconioses, would be exceedingly difficult to treat, and lung damage would likely progress even after reduction or elimination of further LD exposure^66,76^. The overall prognosis for well-studied occupational lung diseases is poor. In the case of silicosis, acute forms of the disease result in increasing functional impairment and death within several years^77^. In accelerated and chronic forms, progressive reduction in lung function often results in death over one to two decades, often as a result of secondary complications, such as infection^29^. In acute silicosis following high-dose exposure, symptoms may begin as soon as several weeks following the event. Standard treatment for these disorders is supportive, consisting of strict avoidance of further exposure, cessation of tobacco use, vaccination against common infectious agents, supplemental oxygen, and bronchodilators as needed. Lung transplantation is the only known cure^78,79^. Experimental therapies targeted at the inflammatory pathways propagating the disease have been explored, most commonly including short or long-term courses of systemic glucocorticoids. These have met with mixed success, and no randomized controlled trials have demonstrated benefit from any specific therapy. Observational studies have suggested improvement with oral steroid use, though in most reported cases, the improvements in lung function are temporary^65^.

Isolated cases have reported positive outcomes with both IL-1 blockade and whole lung lavage, though these successes have yet to be replicated^80^. It is even less certain which of these treatments would be effective in any toxicity arising from LD exposure, as the inflammatory cascades may vary from those of known pneumoconioses. While the full array of therapies would be available in a terrestrial environment after return from an extended lunar mission, the development of lung disease during a lunar stay lasting six months or longer would necessitate a return to Earth to reduce additional LD exposure, so long as the astronaut is functionally able to do so. Until such a return is possible, symptoms would be managed with the limited medical supplies available, likely including supplemental oxygen, bronchodilators, and potentially oral corticosteroids. Ideally, subclinical disease would be detected first using functional or radiological screening, with return to Earth prior to the development of symptoms or significant impairment.

## Future outlook and summary

LD comprises a substantial proportion of the material found on the surface of the Moon, and despite the proximity of Earth’s natural satellite and multiple manned missions to its surface, we understand only the basics of the possible health effects of exposure to this material. Manned and unmanned sample return missions have allowed for detailed characterization of the size and composition LD; additionally, the Apollo astronauts’ brief contacts with LD have provided limited but valuable evidence regarding the immediate symptoms of acute exposure, including both respiratory and ocular irritation. These multi-day exposures, though significant enough to impair performance without countermeasures in place, do not appear to have led to any long-term consequences. To better quantify the risks to 6-month lunar surface missions, the NASA-formed LADTAG studies investigated the inhalational and ocular toxicities of native LD in vitro and in vivo with animal models and determined a PEL for astronauts on any such mission. The results of these experiments suggest the severity of LD pulmonary toxicity to be less significant that than of quartz, a common terrestrial workplace dust responsible for the development of silicosis. However, deleterious effects were still observed at moderate to high concentrations, and the full spectrum of possible other end-organ toxicities was not explored in depth. Further study is needed to delineate the extent of this chronic toxicity and quantify these risks, especially as future lunar outposts may be located in areas with geology different from those of the Apollo landing sites. Finally, new investigations into episodic high-dose LD exposure, such as might occur around suit doffing, are required to better describe any potential disease analog to acute silicosis that would produce in-mission rather than post-mission illness.

Though much foundational work has been accomplished regarding the toxicity of LD, many questions remain unanswered with respect to the possible countermeasures to be implemented. Numerous areas of future study will provide key information for the development of procedures and technologies to better limit and combat LD toxicity, including screening, prevention, diagnostics, and treatment. Depending on mission length, these countermeasures will need to be implemented pre-, in-, and post-flight. Technologies for reducing the contamination of living modules with dust, such as electrostatically repulsive surfaces or suitports, combined with dust removal techniques such as vacuum devices with HEPA and magnetic filters, will help to maintain dust concentrations below the PEL. Intra-habitat LD concentration monitoring will be critical to ensure compliance with these limits, with additional efforts necessary to clean clothing and other surfaces as required. Pulmonary and cardiovascular monitoring may also be necessary, with physical lung exams, diagnostic imaging, and point of care spirometry or sputum analysis all potentially playing roles in ensuring the early detection of any heart or lung pathology. Depending on the end organ effects observed, treatment is likely to be even more challenging in the space environment, though readily available medications such as inhaled beta agonists and supplemental oxygen may be used for respiratory symptom control. While the unknowns of any potential LD-associated pneumoconiosis or other chronic illness are still immense in number, thoughtful pre-flight consideration of and in- and post-flight active monitoring for adverse health effects will provide invaluable data and enable improved countermeasures to target these complications of exposure in the future.

### Reporting summary

Further information on research design is available in the Nature Research Reporting Summary linked to this article.

## Supplementary information

Reporting Summary

Permission from NASA for Use of Figures

## Data Availability

Data sharing not applicable to this article as no datasets were generated or analyzed during the current study other than those published in the references.
