# Supplementary material for: Overview of lunar dust toxicity risk
Source: NPJ Microgravity. 2022 Dec 2;8:55. doi: 10.1038/s41526-022-00244-1 (PMC9718825; doi:10.1038/s41526-022-00244-1)
Supplement: Supplementary file 2 — Permission from NASA for Use of Figures [file 41526_2022_244_MOESM2_ESM.docx]

Explicit permission via e-mail from NASA for use of three figures included in the paper:

Figure 2:

**From:**Michael Stephen Pohlen <pohlen@stanford.edu>
**Date:**Friday, July 3, 2020 at 8:37 AM
**To:**"Pitman, Gwen (HQ-NG000)[MORI ASSOCIATES INC]" <gwen.pitman-1@nasa.gov>
**Subject:**Re: [EXTERNAL] Permission to Use Two NASA Published Photos

Thank you, Ms. Pitman. That all sounds good. I’m sorry to bother again, but one final image I wish to obtain permission for, to which I assume the same regulations and rules apply:

<https://spacephysics.msfc.nasa.gov/science/dusty/pictures/dustgrains.jpg>

From the webpage:

<https://spacephysics.msfc.nasa.gov/science/dusty/index.html>

Thanks much,

Michael Pohlen

Begin forwarded message:

**From:**"Pitman, Gwen (HQ-NG000)[MORI ASSOCIATES INC]" <gwen.pitman-1@nasa.gov>

**Subject: Re: [EXTERNAL] Permission to Use Two NASA Published Photos**

**Date:**July 6, 2020 at 6:34:13 AM PDT

**To:**Michael Stephen Pohlen <pohlen@stanford.edu>

Good morning,

Yes, it would be alright to use the image in the paper.

Have a great day!

Gwen Pitman

--------------------------------------------------------------------------------------------------------------------------------------

Figures 1 and 3:

On Jul 1, 2020, at 8:07 AM, Pitman, Gwen (HQ-NG000)[MORI ASSOCIATES INC] <[gwen.pitman-1@nasa.gov](mailto:gwen.pitman-1@nasa.gov)> wrote:

Good morning,

NASA has no objection to using the two images, “Footprint on the Moon” and Gene Cernan, in the paper.  NASA’s photographs are in the public domain (except where it is noted) and cannot be copyrighted.   We have no objection to them being used as long as their use does not show product endorsement and they are not used in a misleading manner.  There is no charge for using the image.  For additional guideline information, please go to the following web site:

<http://www.nasa.gov/multimedia/guidelines/index.html>

Have an outstanding day!

Gwen Pitman

NASA Headquarters

Photo Office

**From:**Michael Stephen Pohlen <[pohlen@stanford.edu](mailto:pohlen@stanford.edu)>
**Date:**Tuesday, June 30, 2020 at 5:24 PM
**To:**"Pitman, Gwen (HQ-NG000)[MORI ASSOCIATES INC]" <[gwen.pitman-1@nasa.gov](mailto:gwen.pitman-1@nasa.gov)>
**Subject:**[EXTERNAL] Permission to Use Two NASA Published Photos

Dear Ms. Pitman,

My name is Michael Pohlen and I am a resident physician at Stanford University looking to obtain permission to include two NASA-published photos in an upcoming review paper to be hopefully published in the journal Nature Microgravity. They would obviously be cited and labeled appropriately. It appears that NASA allows for publication in non-commercial material like textbooks even without permission but I wanted to confirm.

The two photos are:

NASA. Apollo 11 PSR. *NASA Science* 41–83 <https://www.nasa.gov/specials/apollo50th/photos.html> (1969).

<image001.png>

NASA. Gene Cernan in the Lunar Module. <https://moon.nasa.gov/resources/89/gene-cernan-in-the-lunar-module/> (1972).

<image002.png>

Thanks much!

Michael Pohlen
